# HUCBC Treatment Improves Cognitive Outcome in Rats With Vascular Dementia

**DOI:** 10.3389/fnagi.2020.00258

**Published:** 2020-08-18

**Authors:** Poornima Venkat, Lauren Culmone, Michael Chopp, Julie Landschoot-Ward, Fengjie Wang, Alex Zacharek, Jieli Chen

**Affiliations:** ^1^Department of Neurology, Henry Ford Hospital, Detroit, MI, United States; ^2^Department of Physics, Oakland University, Rochester, MI, United States

**Keywords:** cognition, human umbilical cord blood cells, multiple microinfarcts, vascular dementia, white matter remodeling

## Abstract

**Background and purpose**: Vascular dementia (VaD) is the second common cause of dementia after Alzheimer’s disease in older people. Yet, there are no FDA approved drugs specifically for VaD. In this study, we have investigated the therapeutic effects of human umbilical cord blood cells (HUCBC) treatment on the cognitive outcome, white matter (WM) integrity, and glymphatic system function in rats subject to a multiple microinfarction (MMI) model of VaD.

**Methods**: Male, retired breeder rats were subjected to the MMI model (800 ± 100 cholesterol crystals/300 μl injected into the internal carotid artery), and 3 days later were treated with phosphate-buffered saline (PBS) or HUCBC (5 × 10^6^, i.v.). Sham rats were included as naïve control. Following a battery of cognitive tests, rats were sacrificed at 28 days after MMI and brains extracted for immunohistochemical evaluation and Western blot analysis. To evaluate the glymphatic function, fluorescent tracers (Texas Red dextran, MW: 3 kD and FITC-dextran, MW: 500 kD) was injected into the cisterna magna over 30 min at 14 days after MMI. Rats (3–4/group/time point) were sacrificed at 30 min, 3 h, and 6 h, and the tracer movement analyzed using laser scanning confocal microscopy.

**Results**: Compared to control MMI rats, HUCBC treated MMI rats exhibit significantly improved short-term memory and long-term memory exhibited by increased discrimination index in novel object recognition task with retention delay of 4 h and improved novel odor recognition task with retention delay of 24 h, respectively. HUCBC treatment also improves spatial learning and memory as measured using the Morris water maze test compared to control MMI rats. HUCBC treatment significantly increases axon and myelin density increases oligodendrocyte and oligodendrocyte progenitor cell number and increases Synaptophysin expression in the brain compared to control MMI rats. HUCBC treatment of MMI in rats significantly improves glymphatic function by reversing MMI induced delay in the penetration of cerebrospinal fluid (CSF) into the brain parenchyma *via* glymphatic pathways and reversing delayed clearance from the brain. HUCBC treatment significantly increases miR-126 expression in serum, aquaporin-4 (AQP4) expression around cerebral vessels, and decreases transforming growth factor-β (TGF-β) protein expression in the brain which may contribute to HUCBC induced improved glymphatic function.

**Conclusions**: HUCBC treatment of an MMI rat model of VaD promotes WM remodeling and improves glymphatic function which together may aid in the improvement of cognitive function and memory. Thus, HUCBC treatment warrants further investigation as a potential therapy for VaD.

## Introduction

Vascular dementia (VaD) is a significant cause of cognitive impairment and accounts for nearly 15–20% of dementia incidence in the United States (Plassman et al., 2007; Wolters and Ikram, 2019). VaD is a complex neurodegenerative disease that is characterized by changes in behavior and a progressive decline in cognitive function including the impairment of executive functions such as thinking, planning, problem-solving, working memory, reasoning, judgment, and the execution of tasks. VaD is frequently present in patients after a stroke or a series of mini-strokes and may result from multiple pathologies. Decreased cerebral blood flow to deep white matter (WM) results in silent, multifocal, micro-infarcts in the brain that in turn cause blood-brain barrier disruption, glymphatic dysfunction, increased inflammation, neuronal loss and cognitive impairment in animals (Rapp et al., 2008b; Wang et al., 2012; Venkat et al., 2017). Improved control of vascular risk factors and treatment of the vascular disease may contribute to a decline in dementia incidence in high-income countries (Satizabal et al., 2016; Wolters and Ikram, 2019). There is a need for approved pharmacological and biological interventions that can directly target and improve underlying VaD pathology.

In the current study, we investigate whether human umbilical cord blood cells (HUCBCs) treatment of VaD improves cognition and memory in rats subjected to a multiple microinfarction (MMI) model of VaD. The MMI model in rodents decreases CBF and induces multiple diffuse cerebral microinfarcts, induces blood-brain abarrier dysfunction, WM injury, impairment of glymphatic waste clearance pathway, widespread reactive gliosis, demyelination, hippocampal neuronal damage and cognitive impairment (Rapp et al., 2008b; Wang et al., 2012, 2017; Venkat et al., 2017; Yu et al., 2019). The glymphatic system is a functional waste clearance system that facilitates the removal of interstitial metabolic wastes and neurotoxins from the brain parenchyma (Plog and Nedergaard, 2018; Zhang et al., 2019). This glial-dependent waste clearance mechanism involves cerebrospinal fluid (CSF) penetration into the brain along with periarterial spaces facilitated by perivascular astrocytic aquaporin-4 (AQP4), and interstitial fluid and solute drainage along perivenous channels (Plog and Nedergaard, 2018). AQP4 is an integral membrane pore protein that plays crucial roles in glymphatic waste clearance by facilitating CSF-interstitial fluid exchange and fluid movement throughout the brain (Badaut et al., 2011). Previous studies have reported that loss of AQP4 in AQP4 knockout mice is associated with delayed and suppressed CSF tracer influx compared to wild type mice (Iliff et al., 2012; Mestre et al., 2018). Also, loss of AQP4 exacerbates glymphatic dysfunction, increases amyloid-β (Aβ) accumulation and increases cognitive deficits in Alzheimer’s disease (AD) mice (Xu et al., 2015). Transforming growth factor (TGF-β) is a multifunctional cytokine which can contribute to glymphatic dysfunction by causing vascular changes including the hardening of cerebral vessels (Howe et al., 2019). The hardening of vessels in the brain can lead to a reduction in CSF flow and this can hinder overall waste clearance from the brain parenchyma (Howe et al., 2019). In aging, AD, stroke, diabetes and an MMI model of VaD, impaired glymphatic functioning results in the accumulation of metabolic waste and neurotoxins in the brain which can impair brain homeostasis, injure brain cells and lead to cognitive impairment (Iliff et al., 2012; Gaberel et al., 2014; Kress et al., 2014; Jiang et al., 2017; Wang et al., 2017). Thus, the glymphatic system represents an important target for therapeutic intervention in neurological diseases.

The therapeutic effects of HUCBC transplantation have been previously investigated in several disease models, including stroke (Chen et al., 2001b; Yan et al., 2014, 2015), traumatic brain injury (Lu et al., 2002), and AD (Darlington et al., 2013; Ehrhart et al., 2016). In AD mice, repeated low dose HUCBC administration improves cognitive outcome, decreases glial activation, and Aβ pathology in the brain compared to control mice (Darlington et al., 2013). Also, HUCBC transplantation is safe and well-tolerated in human patients with AD in the Phase 1 clinical trial (Kim et al., 2015). In our previous study, we reported that mice subjected to MMI have significantly decreased miR-126 expression, and mice with endothelial miR-126 knockdown exhibit significant cognitive impairment, WM injury, and glymphatic dysfunction (Yu et al., 2019). MiRs, and particularly miR-126, are emerging as key players in the pathogenesis of vascular damage (Zampetaki et al., 2010). MiR-126, a secreted factor, is one of the most abundant miRs present in endothelial cells and plays integral roles in regulating endothelial cell function, angiogenesis, vascular integrity, and anti-inflammation (Fish et al., 2008; Hu et al., 2015). HUCBC treatment is known to increase miR-126 expression after stroke and contribute to improvement in neurological function in diabetic mice (Chen et al., 2016). Therefore, in this study, we test the therapeutic effects of HUCBC treatment on cognition and memory in rats subjected to an MMI model of VaD and investigate whether HUCBC treatment increases miR-126, improves WM remodeling and glymphatic waste clearance in the brain of retired breeder rats.

## Materials and Methods

All experimental procedures were carried out following the National Institutes of Health (NIH) Guide for the Care and Use of Laboratory Animals and were approved by the Institutional Animal Care and Use Committee (IACUC) of the Henry Ford Health System. This manuscript is prepared following ARRIVE guidelines (Kilkenny et al., 2010).

### Experimental Groups and Timelines

**Figure 1:**
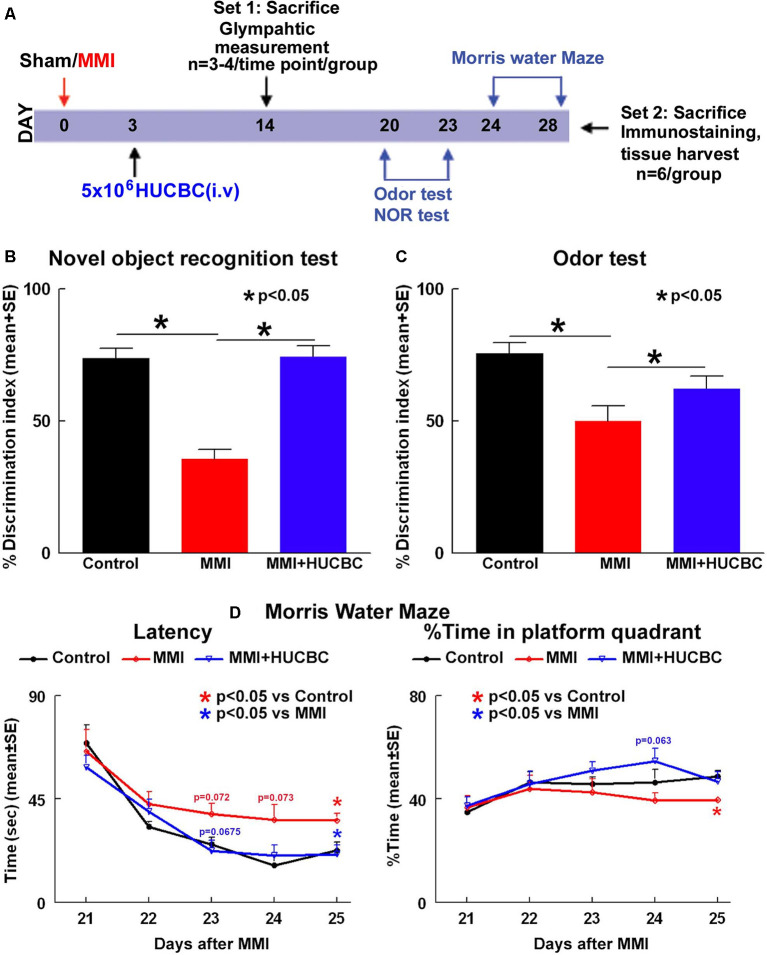
Human umbilical cord blood cells (HUCBC) treatment improves cognitive outcome at 1 month in rats subject to multiple microinfarction (MMI). **(A)** Schematic timeline of the study. Rats were randomized to Sham, MMI, or MMI+HUCBC treatment groups. The MMI+HUCBC group received 5 × 10^6^ HUCBCs administered *via* tail vein injection at 3 days after MMI. Cognitive tests were performed 21–28 days following MMI surgery. HUCBC treatment in retired breeder rats subjected to MMI significantly attenuates VaD-induced **(B)** short-term memory loss (NOR test), **(C)** long-term memory deficits (odor test), and **(D)** spatial learning and memory deficits (MWM test) compared to control MMI rats, *n* = 6/group.

Male, retired breeder (9–12 months old) Wistar rats (Charles River Laboratories, Wilmington, MA, USA) were randomized to one of three groups (*n* = 6/group): (1) sham control; (2) MMI control; and (3) MMI+HUCBC. Rats in the MMI+HUCBC group received 5 × 10^6^ HUCBCs administered *via* tail vein injection at 3 days after MMI. HUCBCs were obtained from Saneron CCEL Therapeutics, Inc., Tampa, FL, USA, a cord blood cell bank. Cryopreserved HUCBCs were rapidly thawed at 37°C and resuspended into 10 ml of 1× phosphate-buffered saline (PBS; VWR) to wash and centrifuged at 100 rpm to pellet. The cell pellet was resuspended in 1× PBS and cell viability and cell quantification were performed using the trypan blue dye exclusion method; cells were diluted in 1× PBS to give the numbers required for the injections. HUCBCs were not labeled before injection. Cognitive tests were performed 21–28 days following MMI surgery, with no overlap in testing. At 28 days after MMI, rats were sacrificed, and brains extracted for immunohistochemical analysis and Western blot analysis. There was no mortality after MMI or treatment in either group. Figure 1A presents a schematic timeline of experiments.

### Randomization and Blinding

Rats were randomly assigned to MMI or MMI+HUCBC treated groups on day 3 after MMI. Investigators performing cognitive tests, immunohistochemical staining, and analysis were blinded to treated groups. ANY-Maze (Stoelting Co.) was used to acquire and analyze cognitive function data.

### MMI Model

Rats were subjected to MMI surgery following previously described methods (Rapp et al., 2008a,b; Wang et al., 2012; Venkat et al., 2017, 2019; Yu et al., 2019). Freshly prepared cholesterol crystals of size 70–100 μm were obtained by serial filtration through 100 μm and 70 μm cell strainers and counted on a hemocytometer. Spontaneous respiration of isoflurane mixed in 2:1 N_2_O:O_2_ mixture was used as an anesthetic and regulated with a modified FLUOTEC 3 Vaporizer (Fraser Harlake). For the entire procedure, rats were placed on a heating pad and body temperature maintained at 37°C. A PE-50 tube was tapered by heating over a flame, connected to a 1 ml syringe filled with cholesterol crystals/saline, and then inserted into the lumen of the internal carotid artery (ICA) through an incision made on the external carotid artery (ECA). A microsurgical clamp was applied to the common carotid artery (CCA) and the cholesterol crystals (800 ± 100 crystals/300 μl saline) were slowly injected into the ICA over a minute. After cholesterol crystal injection, the tube was withdrawn, ECA was permanently ligated with a 4–0 suture, microsurgical clamps removed such that the CCA and ICA remained patent. Rats regained consciousness in their home cages and received routine post-surgical care. Sham control rats underwent a similar surgical procedure and were injected with saline alone.

### Cognitive Testing

(a) Using the novel object recognition (NOR) test, short-term memory was assessed by examining animals’ biases to explore novel objects in their environment. The testing followed previously described methods (Stuart et al., 2013). Briefly, rats were allowed to freely explore two identical objects placed equidistant from the walls of a large box during a 5-min trial phase. Following a retention delay of 4 h, one of the trial objects was randomly replaced with a novel object during the test phase. The trial and test objects were made from hard plastic building blocks and were of comparable sizes but different shapes. Between each trial, the objects and the bin were wiped with water. Exploration times were recorded using ANY-Maze by defining trial and novel objects, exploration zones, and head orientation such that sniffing, biting, licking, pawing, touching with the snout or probing with whiskers <1 cm of the object was included as exploration while sitting on or climbing the object was not included. Discrimination index was defined as the ratio of novel exploration time to total exploration time and a higher discrimination index indicates better short term memory.

(b) The odor test has been described in detail previously (Spinetta et al., 2008) and was used to evaluate long-term memory based on the animal’s preference for novel scents. One week before the test, 2.5 cm wooden beads^1^ were placed in the home cages of two sets of donor rats to build up rat-specific odors, which were later used as novel odors N1 and N2. The test spans 3 days and includes a habituation phase, a trial phase, and a test phase. The habituation phase lasted 24-h during which rats were removed from social housing and placed in a single housing. Each cage had four beads placed in them for familiarization and to serve as a familiar odor (F). On day 2, before the trial phase, all 4 F beads were removed from the cages. The trial phase consisted of 3 1-min trials during which 3 F beads and 1 novel odor bead (N1) were placed in the center of the cage. In each trial, the N1 bead was placed in a new position to minimize spatial cues. An inter-trial interval of 1 min, during which the cage had no beads was used to minimize olfactory adaptation. The test phase occurred after a 24-h retention delay. During the 1-min test phase, four beads (N1, N2, and 2 F beads) were placed in the center of the cage. A four-choice procedure was used for assessing relative odor preference as this has been shown to greatly increase sensitivity and reliability when compared to a two-choice procedure (Spinetta et al., 2008). Time spent exploring (sniffing, licking, biting) each bead was recorded. Discrimination index defined as the ratio between time spent exploring N2 to the total exploration time was used to evaluate long-term memory.

(c) The Morris water maze (MWM) test (Darwish et al., 2012) evaluates hippocampus based spatial and visual learning and memory. The MWM test spans 5 days and consists of four 90-second trials per day. A dark-colored tank was halfway filled with water and virtually subdivided into four equal quadrants [northeast (NE), northwest, southeast, and southwest]. A clear, transparent platform was submerged in the NE quadrant and for every four trials, the platform was randomly moved within the NE quadrant. Rats were introduced into the pool facing the wall from one of four pre-designated starting points and allowed to swim freely for 90 s or until it reached the platform. Data collection including time spent in the target quadrant (NE) and escape latency i.e., time to reach the hidden platform was performed using ANY-Maze software and data were averaged over four trials for each day to yield a single value for each rat/day.

### Glymphatic Measurement

An additional set of sham, MMI, and MMI+HUCBC rats (*n* = 3–4 rats/time point) were employed to evaluate glymphatic function. Tracers including Texas Red conjugated dextran (MW: 3 kDa, Invitrogen) and FITC conjugated dextran (MW: 500 kDa, Invitrogen) were diluted to 1% in artificial CSF. At 2 weeks after MMI, 100 μl of 1% fluorescent tracers at a 1:1 ratio were slowly injected over 30 min using a syringe pump into the cisterna magna, as previously described (Yang et al., 2013; Venkat et al., 2017). Rats were sacrificed at 30 min, 3 h, and 6 h from the beginning of infusion and transcardially perfused with saline and 4% paraformaldehyde. Vibratome sections of brain coronal sections (80 μm thick) were cut and Laser scanning confocal microscopy was used to evaluate tracer intensity in brain sections.

### Histological and Immunohistochemical Assessment

Rats were deeply anesthetized by intraperitoneal injection of ketamine (87 mg/Kg) and Xylazine (13 mg/Kg) which was followed by transcardial perfusion with 0.9% saline using a Simon Varistaltic Pump. For each rat, a section of the brain (frontal cortex) tissue was snapped frozen in liquid nitrogen and stored at −80°C for western blot analysis. The rest of the brain was immersion fixed in 4% paraformaldehyde for 48 h after which the brains were embedded in paraffin and seven equally spaced coronal blocks (~2 mm) cut using a rat brain matrix, as previously described (Defazio et al., 2014). A series of adjacent 6 μm thick C sections were cut and employed for various staining. Brain coronal tissue sections were prepared and an antibody against AQP4 (Millipore, 1:1,000) was employed to assess water channel dysfunction. Antibodies against APC (oligodendrocyte marker, Genway, 1:20), NG2 (oligodendrocyte progenitor cell marker, MilliporeSigma, 1:400), Synaptophysin (synaptic protein, Abcam, 1:400) were also used. Bielschowsky silver was used to stain axons and Luxol fast blue was used to stain myelin. An antibody against amyloid-β 1–42 (amyloid-β 1–42, Abcam, 1:100) was used to assess amyloid-β levels. Similar procedures without the addition of primary antibodies were used as controls.

### Quantification Analysis

As WM/axonal damage was observed in both hemispheres, eight fields of view consisting of corpus callosum (CC), cortex, striatum, and hippocampus were digitized for each brain section under a 20× or 40× objective (Olympus) using a color video camera interfaced with MCID image analysis system (Imaging Research). Positive cell numbers were counted for each field of view or the positively stained area was quantified using a built-in densitometry function in the MCID image analysis system with a density threshold set above unstained. For each animal, data were averaged to yield either percentage positive area or number of positive cells/mm^2^.

### MiR-126 Measurement

Samples were lysed and total RNA was extracted using Qiazol reagents and miRNeasy Mini kit (Qiagen), respectively. According to the manufacturer’s protocols, miRNAs were reverse transcribed with the miRNA Reverse Transcription kit (Thermo Fisher Scientific) and PCR amplification was performed with the TaqMan miRNA assay kit (hsa-miR-126-3p, Thermo Fisher Scientific, catalog #4427975, which is specific for mature miRNA sequences) with U6 snRNA as an internal control (Chen et al., 2016). For the qPCR reactions, we used 2 μl of isolated RNA per cDNA reaction and then used 3 μl of cDNA for each PCR reaction.

### Western Blot

Protein was extracted from samples using Trizol (Invitrogen). The BCA kit (Thermo Scientific) was used to measure protein concentration and 40 μg of protein/lane loaded in a 10% SDS PAGE precast gel (Invitrogen). The gel was placed in a tank where 120 volts of electrical current was run for approximately one and a half hours. The gel was subsequently transferred to a nitrocellulose membrane using the iBlot transfer system (Invitrogen). This membrane was blocked in 2% I-Block (Applied Biosystems) in 1× TBS-T for 1 h, and then either β-actin (Abcam, 1:10,000), TGF-β (R&D Sytems, 1:1,000), Amyloid-β 1–40 (MyBioresource, 1:500) or Amyloid-β 1–42 (Abcam, 1:1,000) was used. For Synaptophysin and PSD-95, 30 μg of protein/lane was loaded, and either GAPDH (Abcam, 1:5,000), PSD-95 (Cell signaling, 1:750) or Synaptophysin (Chemicon, 1:2,000) were used. Secondary antibodies (anti-mouse, Jackson ImmunoResearch) were added at 1:1,000 dilution in 2% I-Block in 1× TBS-T at room temperature. The membranes were washed with 1× TBS-T, and then Luminol Reagent (Santa Cruz) was added. The membranes were then developed using a FluorChem E Imager system (ProteinSimple). Grayscale images were analyzed using ImageJ and normalized to β-actin or GAPDH.

### Statistical Analysis

Repeated measure analysis of variance (ANOVA) was used to test group differences in the water maze test. *Post hoc* analysis was performed, and p-values were adjusted following Tukey multiple comparison testing. Unpaired 2-tailed Student *t*-test was used for comparison of two groups with the use of GraphPad Prism 8. *P* < 0.05 was considered statistically significant.

## Results

### HUCBC Treatment Improves Cognition and Memory in Rats Subjected to MMI

To test whether treatment of MMI with HUCBCs improves cognition and memory in rats, a battery of cognitive tests was performed 21–28 days after MMI. Data in Figures 1B–D show that rats subjected to MMI and treated with HUCBCs exhibit significantly higher discrimination index in NOR task indicating improved short-term memory, higher discrimination index in odor test indicating improved long-term memory, and decreased latency in MWM test indicating improved spatial learning and memory compared to MMI control rats.

### HUCBC Treatment Promotes Glymphatic Clearance in Rats Subjected to MMI

**Figure 2:**
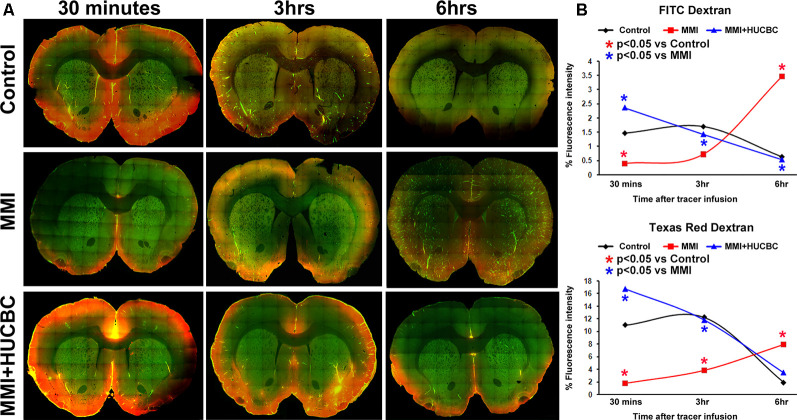
HUCBC treatment improves glymphatic function at 14 days after MMI. In an additional set of sham, MMI, and MMI+HUCBC rats, at 14 days after MMI, 100 μl of 1% fluorescent tracers at a 1:1 ratio (3 kDa Texas Red conjugated dextran and 500 kDa FITC conjugated dextran) was slowly injected into the cisterna magna over 30 min. To analyze tracer movement, rats (*n* = 3–4/time point/group) were sacrificed at 30 min, 3 h, and 6 h after tracer infusion and perfusion fixed. **(A)** Representative images at 30 mins, 3 h, and 6 h after tracer infusion in Sham, MMI, and MMI+HUCBC treated groups. Whole-brain coronal sections (80 μm thick) were imaged under a 10× objective with tiling/image stitching using a laser scanning confocal microscope. **(B)** Tracer fluorescence intensities were quantified using the MCID image analysis system. Compared to the sham group, rats subjected to MMI exhibit a significant delay in tracer influx indicated by low tracer intensities at 30 mins and persisting accumulation with poor clearance from brain parenchyma indicated by high fluorescent intensities at 6 h after tracer infusion. HUCBC treatment significantly improves glymphatic function and reverses MMI induced delay in cerebrospinal fluid (CSF) penetration into the brain parenchyma and clearance from the brain *via* perivascular pathways.

Glymphatic dysfunction has been implicated to induce WM damage and cognitive decline in rats subject to MMI, stroke, and other neurological diseases (Yan et al., 2015). Thus, we tested if HUCBC treatment improves glymphatic function in an additional set of sham, MMI, and MMI+HUCBC rats. At 14 days after MMI, fluorescent tracers (3 kDa Texas Red conjugated dextran and 500 kDa FITC conjugated dextran) were injected into the cisterna magna. To analyze tracer movement, rats (*n* = 3–4/time point/group) were sacrificed at 30 min, 3 h, and 6 h after tracer infusion and perfusion fixed. Tracer fluorescence intensities were quantified in whole-brain coronal sections using laser scanning confocal microscopy. Data in Figures 2A,B indicate that compared to the sham group, rats subjected to MMI exhibit a significant delay in tracer influx indicated by low tracer intensities at 30 mins and persisting tracer accumulation with poor clearance from brain parenchyma indicated by high fluorescent intensities at 6 h after tracer infusion. HUCBC treatment significantly improves glymphatic function and reverses MMI induced delay in CSF penetration into the brain parenchyma and clearance from the brain *via* perivascular pathways.

**Figure 3:**
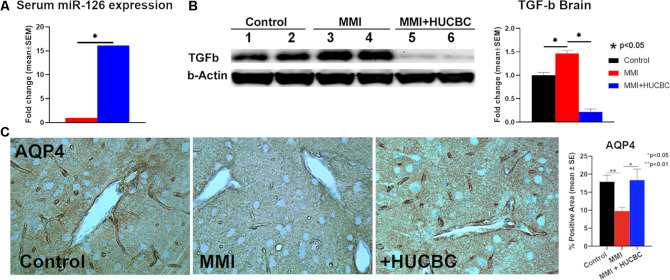
HUCBC treatment increases serum miR-126, increases AQP-4 around cerebral blood vessels and decreases brain TGF-β at 1 month in rats subject to MMI. **(A)** HUCBC treatment significantly increases serum miR-126 expression compared to the control MMI rats, *n* = 6/group. **(B)** Western blot assay showing that HUCBC treatment significantly decreases brain TGF-β protein expression compared to control MMI rats evaluated using Western blot analysis. **(C)** HUCBC treatment significantly increases AQP4 expression around cerebral blood vessels compared to the control MMI rats, *n* = 6/group.

We have previously found that mice subjected to MMI have significantly decreased miR-126 expression which contributes to cognitive impairment, WM injury, and glymphatic dysfunction (Yu et al., 2019). Therefore, we measured serum miR-126 expression and found that HUCBC treatment significantly increases serum miR-126 expression compared to the control MMI rats (Figure 3A). AQP4 expression around cerebral blood vessels is closely related to the proper functioning of the glymphatic system and mice lacking AQP-4 exhibit delayed CSF influx *via* the glymphatic pathways and approximately a 70% reduction in interstitial solute clearance (Iliff et al., 2012; Venkat et al., 2017). Therefore, we evaluated AQP4 expression around cerebral blood vessels and found that HUCBC treatment of MMI significantly increases AQP4 expression compared to control MMI rats which may contribute to improved glymphatic function (Figure 3C). In addition to water channel dysfunction, vascular changes such as hardening of cerebral vessels caused by increased TGF-β may also contribute to the impaired glymphatic function. We found that HUCBC treatment of MMI significantly decreases brain TGF-β expression compared to control MMI rats which may contribute to improved glymphatic function (Figure 3B).

### HUCBC Treatment Decreases WM Injury in Rats Subjected to MMI

**Figure 4:**
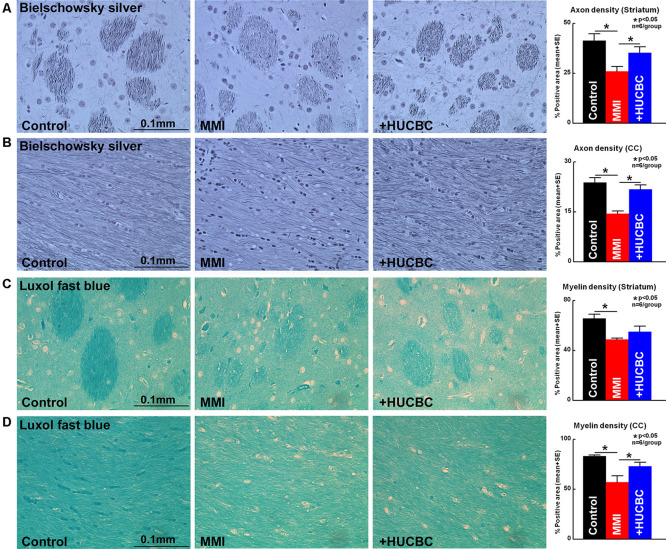
HUCBC treatment improves axon and myelin density in rats subject to MMI. Treatment of MMI with HUCBCs improves white matter (WM) remodeling indicated by significantly increased **(A,B)** axon density and **(C,D)** myelin density in the WM tracts of the corpus callosum and WM bundles of the striatum compared to control MMI rats, *n* = 6/group.

**Figure 5:**
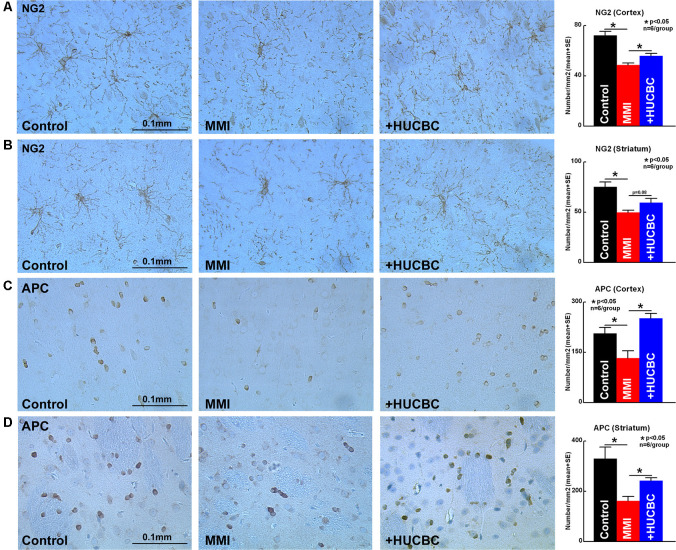
HUCBC treatment improves oligodendrogenesis in rats subject to MMI. Treatment of MMI with HUCBCs improves oligodendrogenesis indicated by a significantly higher number of **(A,B)** OPC and **(C,D)** oligodendrocytes in the cortex and striatum compared to control MMI rats, *n* = 6/group.

WM injury is characteristic of VaD and is associated with cognitive decline in patients as well as in experimental animal models of dementia (Filley and Fields, 2016). MMI rats treated with HUCBCs exhibit significantly increased axon density (Figures 4A,B, Bielschowsky Silver) and myelin density (Figures 4C,D, Luxol Fast Blue) in WM tracts in the corpus callosum and WM bundles in the striatum, as well as significantly increased number of oligodendrocyte progenitor cell (Figures 5A,B, NG2) and oligodendrocytes (Figures 5C,D, APC) in the cortex and striatum when compared to control MMI rats.

### HUCBC Treatment Increases Synaptophysin Expression in Rats Subjected to MMI

**Figure 6:**
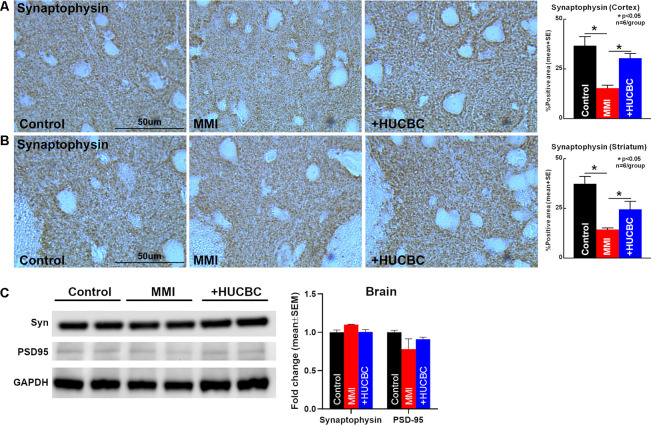
HUCBC treatment improves synaptic vesicle protein in rats subject to MMI. **(A,B)** HUCBC treatment in rats subject to MMI significantly increases the expression of Synaptophysin, a major synaptic vesicle protein, in the cortex as well as striatum compared to control MMI rats, *n* = 6/group. **(C)** Western blot showing Synaptophysin and PSD-95 expression in the brain of sham, MMI, and MMI+HUCBC rats. MMI does not alter cortical brain levels of Synaptophysin or PSD-95 compared to control rats. HUCBC treatment does not alter cortical brain levels of Synaptophysin or PSD-95 compared to MMI rats, *n* = 6/group.

To assess whether HUCBC treatment improves synaptic protein expression in the brain of rats subjected to MMI, we measured the expression of Synaptophysin, the major integral membrane glycoprotein present in neuronal synaptic vesicles (Clare et al., 2010). HUCBC treatment of MMI in rats significantly increases Synaptophysin protein expression in the cortex and striatum compared to control MMI rats (Figures 6A,B). However, we could not detect significant differences in Synaptophysin or PSD-95 protein expression in cortical brain tissue using Western blot between Control and MMI or MMI and MMI+HUCBC groups (Figure 6C).

### MMI or HUCBC Treatment Does Not Alter Amyloid-β in Rat Brain

**Figure 7:**
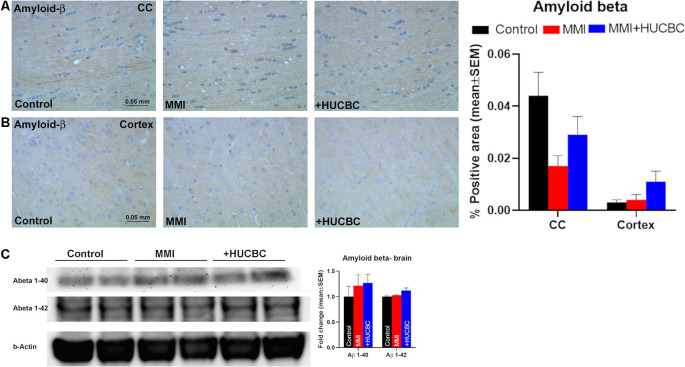
MMI or HUCBC treatment does not alter Amyloid-β in rats subject to MMI. **(A,B)** MMI does not significantly increase Aβ expression in the corpus callosum or cortex compared to control rats. HUCBC treatment does not alter Aβ expression in the corpus callosum or cortex compared to MMI rats, *n* = 6/group. **(C)** Western blot showing Aβ expression in the brain of sham, MMI, and MMI+HUCBC rats. MMI does not increase brain Aβ_1–40_ or Aβ_1–42_ levels compared to control rats and HUCBC treatment does not affect Aβ levels compared to MMI rats, *n* = 6/group.

HUCBC treatment has previously been shown to reduce cerebral and vascular β-amyloid plaques and Aβ_1–40, 42_ peptides in the brain tissue of transgenic AD mice (Nikolic et al., 2008). Therefore, we performed immunostaining to assess amyloid-β levels in Control, MMI, and MMI+HUCBC groups. However, we did not find significant positive staining for amyloid-β in control, MMI, or MMI+HUCBC group (Figures 7A,B). We also tested Aβ expression in the brain of sham, MMI, and MMI+HUCBC rats using Western blot. We did not find a significant increase in Aβ_1–40_ or Aβ_1–42_ levels in the brain of MMI animals compared to control animals and HUCBC treatment did not affect Aβ levels (Figure 7C).

## Discussion

In the current study, we show that HUCBC treatment of VaD in rats improves cognition and memory which may be mediated in concert by enhanced glymphatic clearance, increased miR-126 expression, WM remodeling, and increased synaptic protein expression. Cell therapy continues to be of growing interest as a promising disease-modifying treatment for multiple neurological conditions such as stroke and dementia (Darlington et al., 2015; Yan et al., 2015). HUCBC transplantation has been tested extensively as a treatment for AD (Nikolic et al., 2008; Kim et al., 2015) and stroke (Vendrame et al., 2004; Yan et al., 2014, 2015). There is currently a lack of studies that test the therapeutic benefits of HUCBCs for the treatment of VaD which holds high clinical significance. In this study, we provide evidence for the first time that HUCBC treatment improves cognitive function outcome in MMI-induced VaD model and demonstrates that HUCBC treatment improves glymphatic waste clearance in VaD which may contribute to its therapeutic effects.

HUCBC treatment improves cognitive outcomes in animal models of stroke, traumatic brain injury, and AD (Chen et al., 2001b; Lu et al., 2002; Darlington et al., 2013; Yan et al., 2014, 2015; Ehrhart et al., 2016). In elderly dementia patients, transplantation of HUCBC significantly improves early cognitive functions and daily living activity (He et al., 2017). In prior work, using a transient middle cerebral artery occlusion for stroke in rats, we and others have demonstrated that intravenously administered HUCBCs enter the brain and preferentially migrate to injured brain tissue (Chen et al., 2001b; Vendrame et al., 2004; Xiao et al., 2005). In rodent models of AD, intravenously injected HUCBCs were detected in the brain at both 7 and 30 days after transplantation (Ehrhart et al., 2016). A small percentage of HUCBCs were also distributed in the tissue of peripheral organs such as bone marrow, spleen, liver, and kidney, while they were not detected in the circulation at 24 h after injection (Chen et al., 2001b; Ehrhart et al., 2016). Even when HUCBCs were not detected in the brain following low dose HUCBC intravenous treatment, neuroprotective effects and improvement in neurological function were still observed in the low-dose HUCBC treatment group compared to controls indicating that homing of HUCBC to the brain is not mandatory and HUCBC derived paracrine effects and trophic and growth factors mediate recovery (Borlongan et al., 2004; Xiao et al., 2005). Thus, systemic administration of HUCBCs provides an optimal and viable therapeutic strategy to treat neurodegenerative diseases. VaD is characterized by a decline in cognitive function. Among other symptoms, patients with VaD exhibit memory impairment, disorientation, confusion, anxiety, depression, and attention deficits. Also, patients experience a loss of executive function such as planning, reasoning, organizing thoughts and behavior, and analyzing problems (Venkat et al., 2015). As cognitive impairment is recognized as a key feature in patients with VaD, improvement in memory and cognition is a primary measure for treatment efficacy. In the present study, we employed a battery of cognitive tests to account for the multiple dimensions of impairment seen in patients with VaD. Our data indicate that HUCBC treatment significantly improves short-term memory, long-term memory, and spatial learning and memory as displayed through improved performance in the NOR test, odor test, and MWM respectively when compared to control MMI rats. While HUCBC improves motor function after stroke (Chen et al., 2001b, 2016; Yan et al., 2014, 2015), the MMI model does not induce significant motor deficits in rats (Venkat et al., 2017). In our prior study, we subjected rats of various ages (3–4 months old: young, 6–8 months old: retired breeder and 16–18 months old: aged) to MMI model and performed modified neurological severity score (mNSS) evaluation which tests for motor, sensory, balance and reflex actions and the absence of a tested reflex or abnormal response receives one point (Chen et al., 2001a; Yan et al., 2014). Thus, on a scoring scale of 0–18, a score <6 indicates mild neurological deficits with possibly small to no lesion, a score of 6–12 indicates the presence of substantial neurological deficits with ischemic infarct and score >13 indicates the presence of a very large lesion and predicts poor survival. We found that MMI induces significant motor impairment only in aged rats (16–18 months), while in retired breeder rats there are very slight neurological deficits (average mNSS score of 2.5) on day 1 and low mNSS scores at 21–28 days indicate that motor deficits are unlikely to affect the cognitive outcome (Venkat et al., 2017).

The glymphatic system comprises a brain-wide waste clearance pathway that enables the drainage of CSF and interstitial fluid thereby facilitating the clearance of metabolic waste from the brain parenchyma (Zhang et al., 2019). Dysfunction within this system has been implicated in a variety of conditions including AD, diabetes mellitus, and VaD (Jessen et al., 2015; Venkat et al., 2017). Microinfarcts have been shown to impair the global influx of CSF along the glymphatic pathway and microlesions trap interstitial solutes within the brain parenchyma (Wang et al., 2017). This trapping of solutes and overall impairment of glymphatic function can lead to the aggregation of proteins and inflammation within the brain which can cause neurodegeneration and thus dementia (Wang et al., 2017). AQP4 is mainly present in cells lining the ventricles and in astrocytic end-feet near capillaries. AQP4 is essential in transporting and regulating water movement between cellular, vascular, and ventricular compartments as well as facilitating movement of CSF into the brain, CSF-interstitial fluid exchange, and interstitial solute clearance (Badaut et al., 2011). A widespread loss of AQP4 polarization and overall dysfunction in waste clearance has been found in the aging brain and this reduction in glymphatic clearance has been proposed to contribute to the cognitive decline seen among the elderly (Kress et al., 2014). In our previous study, we have demonstrated that MMI causes a reduction in AQP4 expression around cerebral vessels and induces glymphatic dysfunction with delayed penetration and clearance of CSF *via* paravascular pathways (Venkat et al., 2017; Yu et al., 2019). This glymphatic dysfunction was significantly correlated with reduced cognitive outcome. Our data in the present study indicate that treatment with HUCBCs significantly increases AQP4 expression and improves glymphatic system function at 14 days after MMI. As previously discussed, our data indicate that brain TGF-β expression is increased in MMI rats and subsequently decreased after treatment with HUCBC. TGF-β levels have also been shown to increase in models of stroke and have been implicated in stroke-induced glymphatic dysfunction (Howe et al., 2019). Specifically, increased levels of brain TGF-β expression are associated with an increase in fibronectin within the basement membrane which is an essential regulator of CSF distribution and waste clearance from the brain parenchyma (Engelhardt and Sorokin, 2009; Thomsen et al., 2017; Howe et al., 2018, 2019). Fibrosis of the basement membrane leads to the hardening of vessels and reduces the overall flow of CSF, thus impairing glymphatic waste clearance (Howe et al., 2019). Therefore, an increase in TGF-β may contribute to the glymphatic dysfunction in VaD patients and a reduction in TGF-β levels contributes to the enhancement of glymphatic clearance observed with HUCBC treatment.

WM damage is a common pathology in VaD patients which arises from demyelination and axonal loss due to hypoxia-related damage to OLs (Ihara et al., 2010). WM damage progresses due to the failure of remyelination caused by impaired survival, proliferation, migration, recruitment, and differentiation of OPCs (Maki et al., 2013). Myelin is essential for the coordination and rapid conduction of impulses between cortical regions which are essential for learning and memory (Fields, 2008). As WM facilitates connectivity within the brain which is crucial for the vast array of human cognitive capacities, damage to WM is regarded as a cause of cognitive impairment (Filley and Fields, 2016). A reduction in frontal WM is associated with worse outcomes in working memory tasks (Ehrler et al., 2019) and working and episodic memory dysfunctions also increase with increasing severity of WM lesions (Zeng et al., 2019). HUCBCs have previously been shown to improve OL survival under hypoxia conditions and promote OPC to OL maturation *in vitro* (Hall et al., 2009; Rowe et al., 2010, 2012). In addition, HUCBCs protect striatal WM tracts in animal models of ischemia (Rowe et al., 2010, 2012). Our data show that treatment with HUCBCs significantly increases axon and myelin density in WM tracts in the corpus callosum and WM bundles in the striatum and increases OL and OPC number in the cortex and striatum when compared to untreated MMI rats. Therefore, the promotion of myelination and increase in axon density may be a major contributor to HUCBC induced improvement in cognitive function. Synaptophysin is the most abundant protein present in synaptic vesicles and is an essential factor in synaptic plasticity and memory storage (Clare et al., 2010). It has previously been reported that enhanced synaptic plasticity measured through an increase in synaptophysin expression mediates treatment benefits after stroke (Chen et al., 2010). Using immunohistochemical analysis and imaging under high magnification, we found that HUCBC treatment in MMI rats significantly increases synaptophysin expression compared to control MMI rats. However, we could not detect significant differences in Synaptophysin or PSD-95 protein expression in cortical brain tissue using Western blot between Control and MMI or MMI and MMI+HUCBC groups. The difference between Western blot data and imaging data may be attributed to imaging in specific brain regions compared to using cortical brain tissue for Western blot. Also, the MMI model does not induce wide spread brain damage while synaptophysin is expressed extensively brain wide. Further studies are warranted to test whether HUCBC treatment improves synaptic plasticity in VaD.

In this study, we have primarily examined whether HUCBC treatment improves cognitive outcome and improves VaD pathology but have not tested the underlying molecular mechanisms. MicroRNAs are small, non-coding RNA sequences that are known to regulate many genes, pathways, and biological networks within cells (Kress et al., 2014). MiR-126 is specifically expressed by endothelial cells and primary human endothelial cell lines from veins, arteries, skin, and brain were found to express miR-126 while vascular smooth muscle cells or leukocyte cell lines did not express miR-126 (Harris et al., 2008). Previous studies have analyzed miR-126 level in various murine tissue and found that miR-126 is enriched in tissues with a high vascular component such as heart and lung and expressed at lower levels in the brain, liver, and kidney (Landgraf et al., 2007; Harris et al., 2008; Wang et al., 2008). MiR-126 is significantly decreased in the serum and brain of various animal models of VaD, stroke, and diabetic stroke as well as in cellular models of ischemia/reperfusion (Venkat et al., 2019; Yu et al., 2019; Pan et al., 2020). In a model of transient ischemia, miR-126 was found to be significantly decreased in key brain regions such as the hippocampus and cerebral cortex compared to controls, with the lowest expression in these regions occurring at 24 h after stroke (Xiao et al., 2020). In humans with ischemic stroke, circulating miR-126 is significantly decreased compared to healthy subjects starting at 24 h and lasting until 24 weeks after stroke onset (Long et al., 2013). Serum miR-126 is also decreased in mild, moderate, and severe AD patients compared to healthy controls (Guo et al., 2017). Whether miR-126 expression is altered in human VaD brain is yet to be investigated. The increasing miR-126 expression has been shown to decrease infarct volume, WM and vascular damage, inflammation, and BBB leakage which translates to better cognitive outcomes in dementia and stroke (Venkat et al., 2019; Yu et al., 2019; Pan et al., 2020). Previously, we have found that mice subjected to MMI have significantly decreased miR-126 expression which may play a key role in mediating cognitive impairment after MMI (Yu et al., 2019). Also, mice with endothelial miR-126 knockdown exhibited significant cognitive dysfunction, reduced CBF, WM rarefaction including decreased myelin and axon density, increased inflammation and glial activation, and significant glymphatic dysfunction (Yu et al., 2019). We have previously demonstrated that HUCBC treatment increases miR-126 expression and improves functional outcome, promotes WM remodeling, and reduces inflammation after stroke in diabetic mice (Chen et al., 2016). Therefore, we measured serum miR-26 expression and found that the HUCBC treatment of MMI rats significantly increases serum miR-126 expression compared to the control MMI rats. Thus, it is likely that in MMI as well, HUCBC treatment-induced therapeutic effects are regulated at least in part by increasing miR-126 expression and future studies are warranted.

## Limitation

In humans, as well as rodents, cognitive outcome deteriorates with increasing age (Goldman et al., 1992; Park et al., 2007). It is important to consider age as a factor and employ aged animals in studies investigating mechanisms and therapeutics for VaD. The MMI model effectively induces cognitive deficits in middle-aged rats but not in young adult rats (Rapp et al., 2008b). However, our previous study found that aged rats (16–18 months) exhibit age-induced cognitive deficits, and MMI in aged rats induces significant neurological deficits that can potentially interfere with cognitive test outcomes (Venkat et al., 2017, 2019). Although aged-MMI rats have significant cognitive deficits compared to aged control, aged-MMI induced additional cognitive deficits were not detected using the water maze test compared to aged-control rats (Venkat et al., 2019). In addition to having worse outcomes after stroke than men fluctuations in endogenous estrogens levels can also affect the female brain (Bushnell et al., 2006; Lobo, 2007). Sex should also be considered as a factor that may regulate the association between depressive symptoms and WM lesions (Kirton et al., 2014). In this study, we have only tested the HUCBC effect in male middle-aged rats and future studies are warranted to test efficacy in female middle-aged rats. In the current study, we demonstrate using immunohistochemical analysis that HUCBC treatment of MMI significantly increases axon and myelin density in the WM of the corpus callosum and striatum and increases OPC and oligodendrocyte numbers in the cortex and striatum. However, we did not study the ultrastructure of myelin using Electron microscopy, and further studies are warranted. We have previously demonstrated the therapeutic potential of HUCBC in the treatment of stroke with co-morbid diabetes (Yan et al., 2014, 2015). Also, treatment with HUCBC-derived mesenchymal stem cells prevents inflammation, apoptosis, and remodeling in lung and heart tissue in a model of pulmonary hypertension (Kim et al., 2016). Since hypertension and diabetes are pertinent risk factors for VaD and patients often have co-morbidities, HUCBCs likely have therapeutic potential to treat this population, and future studies are warranted.

## Conclusions

HUCBC treatment of an MMI rat model of VaD promotes WM remodeling, increases serum miR-126 expression, and improves glymphatic function which in concert may contribute to the recovery in the cognitive outcome. Thus, HUCBC treatment warrants further investigation as a potential therapy for VaD.

## Data Availability Statement

All datasets presented in this study are included in the article.

## Ethics Statement

The animal study was reviewed and approved by Institutional Animal Care and Use Committee of Henry Ford Health System.

## Author Contributions

PV performed experiments, analyzed data, and wrote the manuscript. LC performed experiments and wrote the manuscript. AZ, FW, and JL-W performed experiments. MC and JC were involved in experimental design, made critical revisions, and gave final approval of the manuscript.

## Conflict of Interest

The authors declare that the research was conducted in the absence of any commercial or financial relationships that could be construed as a potential conflict of interest.

## Footnotes

^1^https://www.craftparts.com
